# Singapore Success: New Model Helps Forecast Dengue Outbreaks

**DOI:** 10.1289/ehp.124-A167

**Published:** 2016-09-01

**Authors:** Nate Seltenrich

**Affiliations:** Nate Seltenrich covers science and the environment from Petaluma, CA. His work has appeared in *High Country News*, *Sierra*, *Yale Environment 360*, *Earth Island Journal*, and other regional and national publications.

The island city-state of Singapore reported a record 2,441 cases of dengue fever in January 2016,^1^ triggered by all-time-high temperatures in the preceding weeks.^2^ ^,^ ^3^ Singapore is a potential hotbed for the widespread disease thanks to its tropical climate and highly urbanized environment. But it’s also a leader in mitigating the spread of dengue virus through an advanced mosquito control program,^4^ aided in the past 3 years by a sophisticated model to forecast dengue outbreaks.^5^

“The model allows us to confidently warn the public that there could be an outbreak coming,” says coauthor Lee-Ching Ng, who is director of Singapore’s National Environment Agency. “It’s very difficult to be alert at all times. You get fatigued. The public messaging can’t be done all the time, [so] the model suggests when to intensify our message or to mobilize the community.”

**Figure:**
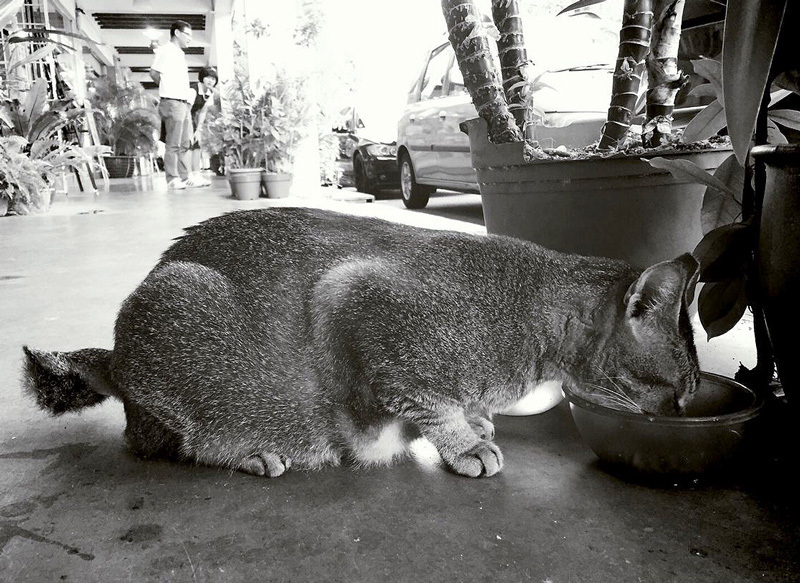
The Singaporean government has launched an advanced mosquito control program to stem the spread of dengue. Along with improved forecasting methods, residents are taught how to look for potential mosquito breeding sites in unexpected places. © Kee Vin Ho/EyeEM

Dengue fever is a flu-like mosquito-borne illness that can develop into a lethal severe form.^6^ The World Health Organization considers it the world’s fastest-growing vector-borne disease, with a reported 30-fold increase in incidence over the last 50 years.^7^ Recent estimates put the number of annual infections as high as 390 million, with 96 million symptomatic cases.^6^

The new forecast model was developed collaboratively by researchers at the National Environment Agency and two local universities. The model uses a state-of-the-art “machine learning” method called least absolute shrinkage and selection operator, or LASSO. Machine learning refers to programs that improve their predictive ability over time (i.e., “learn”) by repeatedly identifying patterns among complex data inputs. This model includes more than 200 variables—including recent dengue fever cases, weekly mosquito surveillance data, population and weather data, and disease seasonality factors—to generate weekly forecasts of dengue activity nationwide for the upcoming 1–12 weeks.

According to Cory Morin, a fellow in the NASA Postdoctoral Program at Marshall Space Flight Center, the quality of a disease forecast degrades as it goes further into the future. “This is true of almost all models, so it certainly does not detract from the success of the [current] study,” he says. “But it is important for understanding why the forecasts are limited to twelve weeks.” Morin was not involved in the study.

The model was first employed in 2013 and quickly demonstrated its potential, forecasting major outbreaks in 2013 and 2014 more than 10 weeks in advance.^5^ With an outbreak on the horizon, the government prepares hospital beds and diagnostic kits, deploys a staff of about 800 for on-the-ground mosquito control and community outreach, and launches a multi-platform campaign to encourage residents to eliminate any stagnant water and apply mosquito repellent, says Ng. The ability to forecast outbreaks provides opportunities for research on the effectiveness of public health and mosquito control interventions; however, no such data are yet available.

One challenge presented by the new model—at least in terms of communicating results to stakeholders—is that it doesn’t show its work. Instead, it delivers a forecast without explaining which factors and variables influenced the outcome. “It acts like a little black box,” says senior author Alex Cook, a statistician with the National University of Singapore. “It takes a big basket of risk factors and within the computer itself decides which combinations of those it will use. It’s almost impossible to interpret cause and effect.”

A potential shortcoming is that the model isn’t tailored to deliver the sort of daily, location-specific results that are most useful to front-line critical responders working across larger regions. “It’s very important for most public health workers to know which area will have a higher risk,” says Ta-Chien Chan, a researcher at Taiwan’s Academia Sinica who was not involved in the study. “They have to know where, in real time.”

However, Chan adds, in the context of Singapore’s relatively small, densely populated landmass and established dengue control program, the advantage of this model is that it provides an early warning signal. “It can also provide insight on the trend—how the epidemic will proceed,” he says. “This is also very important information for preparedness.”

The Singaporean government continues to work toward even longer-term, more accurate dengue forecast models. Ng says, “We are improving the accuracy of the model and increasing the spatial resolution to risk-stratify areas for targeted response.”
